# Innovate emergency governance mechanism of urban communities in response to major public health events: A qualitative study from multiple principals in Guangzhou, China

**DOI:** 10.3389/fpubh.2023.1008378

**Published:** 2023-03-02

**Authors:** Liting Zhou, Fei Ouyang

**Affiliations:** School of Urban Culture, South China Normal University, Guangzhou, China

**Keywords:** urban community, major public health events, emergency governance mechanism, multiple principals, Guangzhou city

## Abstract

Since the end of 2019, the sudden outbreak of COVID-19 has challenged the emergency governance systems of various countries. As the cornerstone of national governance, China's community emergency governance mainly adopts top-down organizational mobilization and rapid response, which is typical abnormal governance. In responding to major public health events, China's national system has developed certain advantages in some respects. However, the current pandemic is still serious in many places, and new mutant strains are constantly appearing. Some drawbacks of such system and mechanism are gradually emerging. In the process of preventing and controlling the pandemic, China's urban communities have continuously improved the joint mechanism, and played the role of multiple principals in collaborative and co-governance. The current work of pandemic prevention and control has entered a period of normalization. What is the collaborative mechanism of multiple principals (Subdistrict headquarter, Community committee, Owners' committee, Community hospital, Local police station, Property management company, etc.) in urban communities participating in emergencies and how to seek ways to further improve the mechanism? Therefore, taking the community practice and actions in Guangzhou, China as an example, the present study employed a qualitative design, proposed to better community emergency governance mechanisms from the aspects of preparedness, response, communication and recovery, so as to provide a reference for other grassroots organizations.

## 1. Introduction

Under the multiple promotion and superimposed pressure of urbanization, industrialization and informatization in China's modernization process, emergencies such as natural disasters, accident disasters, public health events and social security events have continuously disturbed the community. Chinese society has inevitably entered a holistic risk society, which puts forward new requirements for capacity building of community emergency governance (1). Compared with the other three types of emergencies, public health emergencies are more likely to have an all-round impact on social development due to their complex causes, unpredictable, difficult to prevent, rapid spread and wide-ranging impacts. As the “first scene” of public health emergencies, the community plays a fundamental role in the national emergency governance system. Therefore, how to better play the role of community emergency governance in public health emergencies, build a modern emergency governance system and improve the capacity of community response to public health emergencies has gradually become an important topic of academia and governments (2–4).

It is worthwhile to mention that the concept of “community” used in this study is not a residential area in a narrow sense, but a “community” in a broad sense, which is to explain from the social and the government management significance (5): Understanding from the social significant, “community” refers to a regional community of social life, which mainly includes the organizations such as subdistrict headquarters, community committees, owners committees, community hospitals, police stations, property management companies, as well as residents' families and volunteer groups. Understanding from the government management, “community” refers to the administrative area bounded by the jurisdiction of the community committee.

In the context of the normalization of COVID-19 prevention and control, the research on community emergency governance thus has become a new hot spot. Researches related to this topic at home and abroad mainly focus on three aspects: the mode transformation, the technology application, and the capacity assessment of community emergency governance. Among them, Chinese scholars firstly pay attention to the innovation of the governance mode and resilience governance. They not only emphasize that the community is the first scene to respond to emergencies and propose a governance model based on the governance theory, but also emphasize that the governance model of public health emergencies should gradually change from the traditional model of “government prevention” to the “triangular structure” of “coordinated prevention” of government, social organizations and community residents, so as to realize the linkage of governance capabilities (6–8). Secondly, they focused on the research about smart governance and emergency governance of smart community construction, and refined community grid management which is mostly defined as a modern urban management technology in China by dividing the network format of the community, and the population, resources, and daily management of the grassroots society are delimited in a relatively independent and fixed spatial framework, so as to enhance the community management capacity and improve the work efficiency (9–11). Thirdly, they focus on the evaluation of comprehensive emergency response capabilities for natural disasters in urban communities (12, 13), and the assessment of the ability to respond to public health emergencies begun to be noticed after the outbreak of COVID-19 (14, 15). Foreign scholars firstly focus on strengthening the joint participation of stakeholders in responding to major disasters (16–18); Secondly, they mainly focus on the relationship and application of emerging technologies and community emergency management (19–22); Thirdly, they focus on the analysis model and evaluation system of “community resilience” to evaluate the emergency response capabilities (23–26).

However, although the domestic and foreign researches on community emergency governance have achieved a series of achievements, these researches are mostly concentrated in the fields of natural disasters, and the public health research tends to be fragmented (16, 18, 27). In addition, existing researches focus on multiple principals jointly responding to public crises, using Big Data and other technologies to improve urban community emergency governance, and innovating the governance mode and system, but the research on the community public health governance mechanism of multiple principals collaboration has not attracted widespread attention (7, 11, 17, 19, 21). Especially, the community needs to have multiple interactions and exchanges with social system, which is the prerequisite for its real resilience. What's more, the existing research on community emergency governance still lacks the construction of its mechanism from a structured perspective. Under the background of the normalization of pandemic prevention and control, the daily governance in the normal period and the emergency governance in the abnormal period are switched with each other. Therefore, it is necessary to establish the logical governance relationship between the two states from the perspective of the whole life cycle.

In light of the lack of the above researches, how do multiple principals participate in urban community emergencies in the context of major public health events? In addition, what experiences and deficiencies do the multiple principals have in the process of collaborative governance? Based on this, how should we innovate the emergency mechanism on urban community? Therefore, the purpose of this study is to determine the collaborative mechanism of multiple principals in urban communities, analyze its advantages and dilemmas, so as to seek ways to further improve the mechanism of community emergency governance.

Guangzhou, the capital city of Guangdong Province in China, ranks among the top four in terms of GDP in the country and has a permanent population of over 18 million, so it is a typical mega city (28). For the past 20 years, it has experienced the SARS coronavirus epidemic in 2003 and the coronavirus disease 2019 pandemic (29, 30). Guangzhou became the main battlefield in the fight against SARS in 2003. Faced with the sudden epidemic, Guangzhou has gradually paid attention to the important role of community epidemic prevention from hasty challenge to proactive attack, and finally won the victory in the fight against the SARS epidemic (31). Facing the fast-spreading of COVID-19, Guangzhou adhered to the principal of “take rapid prevention and control actions to control the rapid spread of the pandemic” with mobilizing the participation of multiple forces, and achieved miracles of anti-pandemic again and again. For example, since April 8, 2022, a new round of pandemic has occurred in Guangzhou, and various administrative districts of Guangzhou have successively launched nucleic acid testing for all citizens. In just 24 h, 16.664 million samples have been taken (32). As of April 20, the “0408 pandemic” in Guangzhou has achieved phased results, and the spread of the virus has been basically blocked. Due to Guangzhou's outstanding anti-epidemic achievements in responding to the SARS epidemic and the coronavirus disease 2019 pandemic, its anti-epidemic experience and ways are worthy of our learning and reference. Therefore, taking Guangzhou city as an example, we conducted in-depth interviews with multiple principals participating in community emergencies, and combined with second-hand information such as official documents published by the government, to try to innovate the emergency governance mechanism for urban communities to respond to major public health events.

## 2. Materials and methods

### 2.1. Objectives

This study utilized a qualitative research design method to explore the multiple principals and their collaborative governance mechanism that directly related to urban community emergency governance in the context of normalization of pandemic prevention and control. The community is the smallest “cell” of a city and the grassroots governance. Therefore, in response to major public health events such as COVID-19, the main actors in urban communities include: subdistrict pandemic prevention and control headquarters, community committees, community health service centers (community hospitals), owners committees, property management companies, and community police offices, etc. This study mainly uses in-depth interviews to conduct research on the functions and specific work of these grassroots governance organizations and their mutual emergency mechanisms.

### 2.2. Sampling

Qualitative methods are suitable for investigating exploratory questions. Therefore, semi-structured interview method was used to achieve the purposes of this study and describe the perspectives of multiple principals involved in emergency governance in urban communities. The interview questions mainly involved: (1) The responsibilities and functional orientation of the organization, the main staff and organizational structure, and the daily division of labor; (2) Difficulties encountered by each principal when participating in community emergency work in the context of normalization of pandemic prevention and control and the final solutions; (3) What preparations or early warning work does the community usually do in response to emergencies; (4) When the pandemic suddenly comes, how to quickly cooperate with other principals to carry out pandemic prevention and control affairs? This study was conducted from mid-June to mid-July 2022 and involved 28 interviewees, all of whom were members of grassroots organizations in C subdistrict, H administrative district, Guangzhou. C subdistrict has always adhered to the concept of putting life first in the process of pandemic prevention and control. Through rapid emergency response actions, C subdistrict has achieved outstanding results. Especially, some communities in C subdistrict even maintain a record of “zero infection”, so their related grassroots organizations have been awarded the title of “Guangzhou Advanced Group in Fighting against the COVID-19” by the Municipal Government. Therefore, the public health emergency governance experience and specific plans of C subdistrict can provide rich information for this study to the greatest extent, and have certain typicality and reference value (33). Before the in-depth interviews began, we explained the purpose of study and survey method to all participants. On average, each interview takes 30–60 min. After completing 20 interviews, all the audiotaped were transcribed verbatim by the authors, and a total of 66,785 words of interview transcripts were obtained after deleting the contents irrelevant to this study. In order to protect the personal information of the interviewees, this study encodes them to ensure the anonymity and confidentiality of the interviewees. Basic information of the interviewees are shown in Table 1.

**Table 1:** Basic information of interviewees.

**Code**	**Institution**	**Profession**	**Method**	**Date**
S1	Pandemic prevention and control headquarter of C subdistrict	Deputy director	Telephone interview and WeChat interview	2022-06-15
S2		Full-time cadres 1	On-site interview	2022-06-12
S3		Full-time cadres 2		
C1	JX community police office	Police officer	Telephone interview and on-site interview	2022-06-18
C2		Auxiliary police	On-site interview	2022-06-16
H1	Community health service center of C subdistrict (community hospital)	Director	On-site interview and WeChat interview	2022-06-21
H2		Section chief		
N1	JX community committee	Director	On-site interview	2022-06-25
N2	QXG community committee	Director	On-site group interview	2022-06-29
N3		Deputy director		
N4		Full-time staff 1		
N5		Full-time staff 2		
N6		Full-time staff 3	On-site interview and WeChat interview	2022-07-01
N7	CT community committee	Deputy director	Telephone interview and WeChat interview	2022-07-05
N8		Full-time staff	On-site interview	2022-07-03
O1	XT owners committee	Full-time cadre	Telephone interview and On-site interview	2022-07-08
O2		Chief supervisor	On-site group interview	2022-07-15
O3		Director		
O4		Committee member 1		
O5		Committee member 2		
P1	XT property management company	Director	Telephone interview	2022-07-17
P2		Customer service assistant	On-site interview	2022-07-17
P3		Safety management foreman	On-site interview	2022-07-17
P4		Security member 1	On-site interview	2022-07-16
P5		Security member 2	On-site interview	2022-07-16
V1	JX party member voluntary organization	Secretary of grid Party branch	On-site interview	2022-07-19
V2	QXG party member voluntary organization	Member of grid Party branch	On-site interview	2022-07-19
V3	CT party member voluntary organization	Member of grid Party branch	Telephone interview	2022-07-20

The government departments in Guangzhou actively utilized official websites and new media platforms to release relevant emergency governance information during the response to pandemic. In accordance with the principle of “trigonometrical survey”, this paper uses a variety of data sources and collection techniques. Finally, this study mainly collects secondary sources from policies and regulations, notices, news reports and internal documents related to the emergency governance of major public health events through the official websites of government at all levels in Guangzhou, new media platforms and interviewee channels, so as to better understand the emergency governance mechanism and the shortcomings of multiple principals' collaborative governance on urban communities.

### 2.3. Data analysis

In line with principals and processes common to thematic analysis (34), the authors used NVivo software to analyze the interview transcripts of the above 28 respondents. Firstly, read each interview transcript carefully and code the key statements. Secondly, conduct inductive analysis and theme extraction of all coded statements, and identify the common themes and shortcomings of multiple principals participating in urban community emergency governance through systematic comparison of interview texts with different principal, so as to illustrate the convergence of answers among all participants and specificity. Thirdly, explain the themes after induction and highlight phenomena that represent specific but shared views of grassroots governance characteristics. Finally, the authors re-read all the statements of the interview text and discuss the themes and findings by induction until both authors agree on the results of the qualitative analysis.

## 3. Results

**Figure 1:**
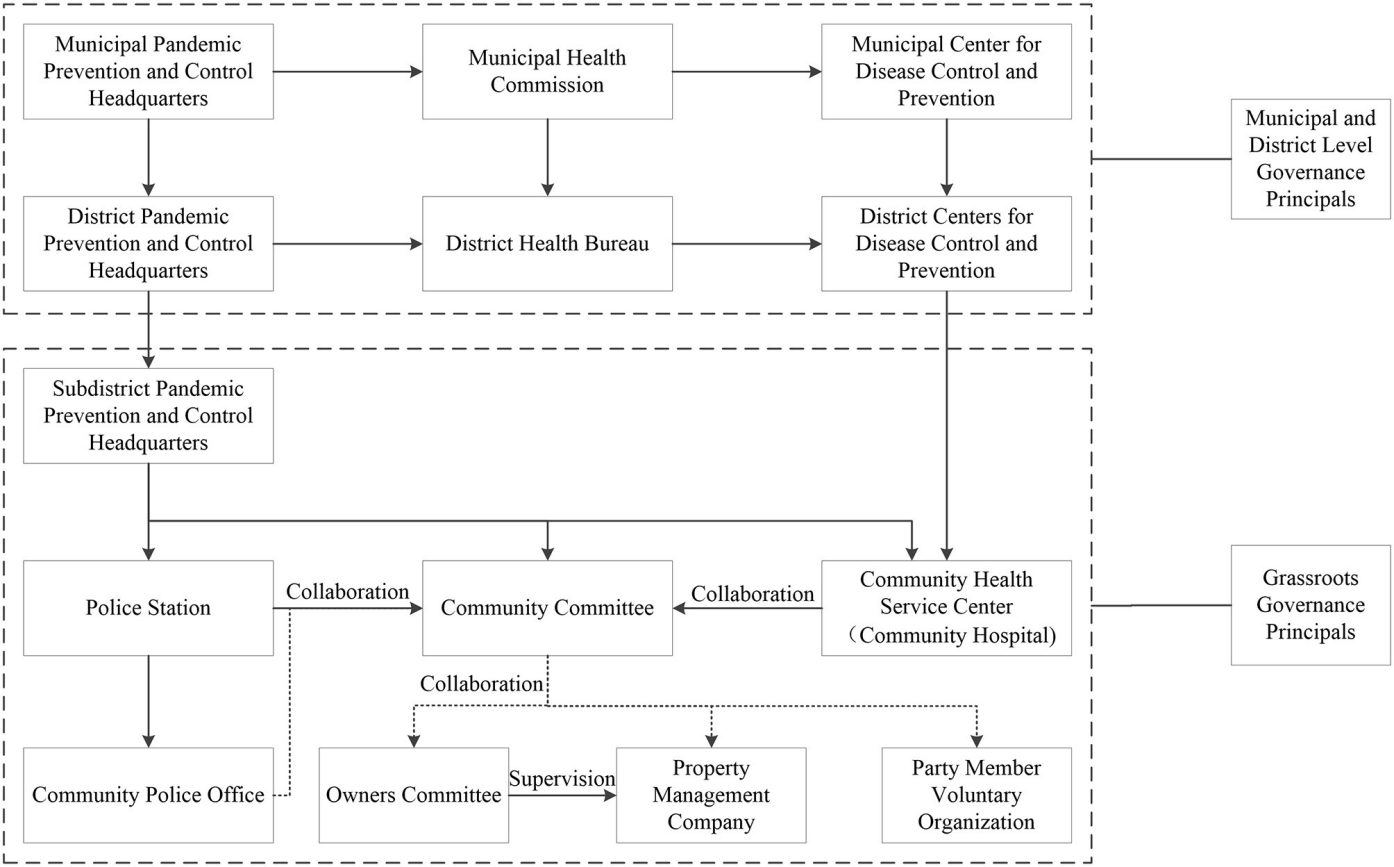
Collaborative governance diagram of grassroots organizations.

Based on the descriptions of the interviewees and official reports, we found that the grassroots organizations in response to major public health events have collaborative governance relationships as shown in Figure 1.

Urban community emergencies are important part of grassroots governance. As shown in Figure 1, a subdistrict usually governs multiple communities, and the subdistrict pandemic prevention and control headquarter is an important part of the subdistrict office. In the process of responding to COVID-19, the subdistrict headquarter is subordinate to the district one, and then the sub district headquarter directs the work of “three-person team”. The so-called “three-person team” is composed of three departments: the police station, the community committee, and the community health service center (community hospital). It is also the smallest emergency unit at present. The responsibilities of the “three-person team” includes: checking the pandemic-related screening list, preliminary screening and control for core personnel, daily health monitoring and sampling for community residents. Among them, the police station mainly provides force to maintain the stability of the scene, the community hospital mainly provides professional medical services. In addition, the pandemic-related screening list is issued by superior public security department, and the “three-person team” is required to verify the pandemic-related situation and urge relevant people to test nucleic acid. The list of core personnel (close contacts, secondary close contacts or other core people) is issued by the district centers for disease control and prevention, and the “three-person team” is required to verify the actual residence of core people and implement control measures in a timely manner according to requirements. If a person who is suspected to be involved in the pandemic or a person with a red health code is found in a public place (the health code is a QR code of personal health information generated after the background review based on the real data reported by citizens through the internet), the executives of public places (subway stations, bus stations, shopping malls, fever clinics, etc.) must directly contact “three-person team” to verify the suspicious situation as soon as possible and timely implement control or deregulation as requested. When there is a suspected or confirmed case, the subdistrict headquarters will be notified by the district headquarters, and then organize the department corresponding to “three-person team”, in order to immediately arrange sufficient personnel to the scene. According to the attribute of the pandemic (single person tested positive for initial sampling/mixed samples from multiple people tested positive/re-positive in cases), the scope of control will be confirmed firstly. And later, the responsible principals (enterprises and institutions or community committees, community hospitals and property management companies etc.) will be contacted to jointly implement the control measures with the police station as a priority.

Subsequently, the “three-person team” cooperates with the centers for disease control and prevention according to the requirements of the superior, and conducts nucleic acid re-testing of positive cases as soon as possible. If the result of re-texting is positive, the designated hospital will arrange closed-loop transfer vehicles to carry infected persons for treatment. Meanwhile, the headquarters will evaluate and delineate high, medium or low risk areas, and the “three-person team” will conduct nucleic acid testing on residents of the buildings/residential areas involved in the pandemic (the frequency of nucleic acid testing will be determined according to the level of the risk area), and get the residents' nucleic acid testing results as soon as possible to confirm whether there are continuing. The centers for disease control and prevention will carry out epidemiological investigations when it is informed of the initial screening of positive cases, and identify core places, core close contact, general close contact, secondary close contact etc., so as to implement accurate and appropriate emergency measures according to the current situation.

### 3.1. Positive themes

#### 3.1.1. Emphasis on pandemic prevention work

Guangzhou takes into account both emergency response to sudden outbreaks and normalized prevention and control. On the one hand, government departments conduct in-depth investigation and traceability of population movements to speed up the identification of risk sites involved in cases, so as to achieve rapid measures to stop the spread of the pandemic. On the other hand, government carries out normalized measures such as nucleic acid testing with solid quality. Due to the fact that Omicron and the recent BA.4, BA.5 mutant strains are easy to make some early infected people difficult to be found, when the Omicron cluster broke out in many places in China at the earlier stage, the virus has been hidden and spread in the community for a period of time, which has brought great difficulties to the tasks of anti-pandemic. Among the various measures to fight the pandemic, nucleic acid testing is the key to quickly discover the source of infection to lock the control target, and then taking measures such as isolation to cut off the transmission route. The past experience proves that nucleic acid testing has played an important role in the successful disposal of clustered outbreaks. With the normalization of prevention and control, the work of grassroots organizations has shifted from treatment to prevention when there is no outbreak. The director of the JX community committee described the daily work in the community:

*The nucleic acid testing we organized recently is made overall arrangements by the municipal, district and subdistrict headquarters. The government of Guangzhou City stipulates that H district must complete sampling of a fixed population every day. For example, the amount of nucleic acid testing that needs to be completed every day in our C subdistrict is 30,000 to 40,000. There are a total of 22 community committees within our jurisdiction. Generally, at least 12 community committees carry out nucleic acid testing every day, and our JX community committee has a daily testing task of 3,600 people*.

The timely detection of infected people can stop the spread of the pandemic as soon as possible through normalized nucleic acid testing for residents, and minimize the impact of the pandemic on society ultimately. The director of the JX community committee views the normalized measures of testing as follows:

*The normalized nucleic acid testing measure reflects the concept of pandemic prevention priority. If there are confirmed cases for treatment, the government will endure heavier expense. Although our daily work is very hard for completing the testing task, we have screened many asymptomatic infections. Every resident is basically screened once or even several times. In this way, cases are screened at an early stage, and their activities are still relatively small, so we can easily block the transmission chain of the virus*.

#### 3.1.2. Mobilize multiple social forces

As the capital of Guangdong Province, Guangzhou has a huge population mobility and a large number of immigrants. When a small-scale epidemic has not yet occurred, the implementation of normalized nucleic acid testing is conducive to improving the sensitivity of monitoring and warning, and discovering potential risks earlier. However, carrying out normalized nucleic acid testing requires a lot of human support, and it is difficult to meet the needs only with community hospitals' personnel. Therefore, the local government makes full use of the market and allows third-party forces to assist hospitals for completing the task *via* financial allocations, which greatly alleviates the problem of insufficient professional medical personnel. At present, Huayin Medical Testing Center and Zhongxin Gene Medical Technology Co., Ltd. are mainly responsible for the normalization of nucleic acid testing in C subdistrict. Deputy Director of CT community committee replied:

*Nucleic acid testing was originally the responsibility of community hospitals. Because there is only one community hospital in our subdistrict, their human resources and capabilities are very limited. They are not only responsible for pandemic prevention and control, home isolation and monitoring, as well as daily basic medical and health services to meet the needs of residents, but also responsible for the normalized nucleic acid testing of residents, then these workers will need to work 24 hours a day. Therefore, they have to cooperate with the third-party agencies to carry out normalized nucleic acid testing work in the form of public service outsourcing, while the community hospital allocates their limited human resources to emergency affairs of “three-person team”*.

In addition to relying on the strength of market principals, C subdistrict also gives full play to the role of “red house” party building brand for mobilizing the community resources of party member volunteers, and cooperates with this service force of social work stations to support various emergency governance in communities. These volunteers are mainly distributed in mass testing or population-wide testing programs, and vaccination, material distribution, information verification positions, effectively helping government and grassroots organizations such as community committees to alleviate the serious shortage of staff. The party group secretary of JX voluntary organization replied:

*During normalized or mass nucleic acid testing, we recruit party member volunteers to assist us in scanning residents' QR codes for entering personal information, and instruct the elderly to operate mobile phones for completing personal registration or open the personal QR code. Party member volunteers in our community can reach up to about fifty people a day*.

The chief supervisor of XT owners committee, who also serves as the secretary of party branch in the community grid replied:

*The party organization of my work unit requires in-service members to report to the community and support this great work, and I am a party cadre managed by the department in my institution, so I take the initiative to participate in the volunteer activities for pandemic prevention and control in our community and serve as a secretary of community grid party branch (voluntary and unpaid). I hope I can do my best to help the community committee finish the task of pandemic prevention and control. Regarding me, the secretary of the grid, is actually more of a volunteer*.

#### 3.1.3. Strengthen humanistic care

The first is to focus on the response for vulnerable groups such as the elderly. Because the immune function of the elderly is weak and most of them have various underlying diseases, once infected by the virus, the risk of severe illness and death is quite high. Therefore, the elderly are the most vulnerable group of people susceptible to the COVID-19. Fortunately, the 2019-nCoV vaccine has a significant effect on preventing the severe illness and death. China's vaccination strategy is to follow high-risk groups, core groups, and finally gradually transition to the elderly over 60 years old at present. By increasing the vaccination rate of the elderly, it can effectively fill in the shortcomings of the immune barrier. Hence, the city's prevention and control work can achieve the initiative. In order to encourage the elderly over 60 to be vaccinated, various subdistricts in Guangzhou adhere to the principal of voluntary vaccination, and mobilize the enthusiasm of the elderly by giving gifts and free shuttle service. Take the vaccination notice for the elderly announced by JX community committee as an example:

*Unvaccinated people over the age of 60 who participate in the health assessment can receive a small gift regardless of whether you meet the requirements for vaccination. Seniors over 60 who inoculate the first shot of vaccine can receive a 100-yuan shopping coupon, a pack of rice and a 168-yuan e-commerce gift coupon card on the spot. If the elderly need to get the first shot of vaccine, they can contact community committee to pick them up*.

The second is to ensure the normal life of special groups. In order to minimize the impact of major public health events on the society, the Information Office of Guangzhou Municipal Government reported: For special places and institutions such as nursing homes and welfare homes, senior high school teachers and students, wholesale markets, freight and express workers, pandemic prevention workers and volunteer groups, the frequency of nucleic acid testing should be increased to promote these places or groups to restore normal working order as soon as possible.

The third is to actively respond to residents' demands. For the control areas affected by the pandemic, H district has established a “four-leader system for service guarantee” consisting of the special class monitor, the grid leader (It is a post for the implementation of grid management in cities in China, and is mainly responsible for the inspection, investigation, and reporting of community management within the grid), the building leader and the floor leader, implemented a “three-person team” and a “double list” system of service objects, and announced a 24-h service hotline for community residents to respond the service needs in a timely manner. The members of the party group in Municipal Affairs Service Data Administration reported at the press conference on July 13, 2022:

*The 12345 Government Service Hotline opened in Guangzhou cooperates closely with 110 Alarm Platform and Psychological Assistance Hotline to establish a collaborative mechanism for special matters, strengthen the 24-hour real-time handling of matters involving high, medium and low risk areas, and coordinate or resolve urgent demands of pregnant women, patients, and infant milk powder etc. Since the beginning of this year, Guangzhou 12345 Hotline has received a total of 41,849 appeals related to the old, the weak, the sick, the disabled, and the pregnant. Then it dispatched 30,148 cases, and answered 11,701 cases directly*.

The fourth is to provide convenient nucleic acid testing services for the masses. For example, the community will provide residents with free testing services every day in the context of the normalization period. In this process, C subdistrict actively explored the combination of “fixed nucleic acid sampling points plus mobile sampling vehicles” for residents, then implemented the measures of “exhaustive testing” and “those who are willing to test are tested” for fine nucleic acid testing. Its mobile sampling vehicles are designed to further facilitate people participating in test nearby and accept it in a relatively comfortable environment. In addition, in view of the actual situation that there are many elderly people in some communities under the jurisdiction of C subdistrict, the sampling point has also set up special channels for the elderly or the disabled, and adopts rapid testing methods such as mixed sampling of “five people in a group” for them, which has greatly shortened the waiting time and truly made it convenient for the residents. Ultimately, the coordination of residents with nucleic acid testing rapidly improved.

### 3.2. Negative themes

#### 3.2.1. Heavy emergency workload

Due to the highly contagious, sudden, and urgent nature of COVID-19, non-contact services are usually used, which imposes high requirements on the number and professional skills of community workers. According to the survey, the community hospital has more than 30 full-time doctors, each community police office has about 1 or 2 auxiliary policemen, and the 22 community committees usually have 5–7 full-time staff, about 15 security guards and varying numbers of party member volunteers in C subdistrict, H district, Guangzhou city, totaling about 536 workers. For the 159.7 thousand residents in C subdistrict, this set of data means that on average 1 community worker needs to provide corresponding services for about 300 residents. Because community workers not only need to carry out the task of “case screening—temperature measurement—hemogram/CT detection for suspected cases—case investigation and reporting—arrangement for confirmed patients to be hospitalized”, which requires a lot of human and material resources, but also need to supervise the property management company carrying out sanitation and environmental disinfection or sterilization. Such as organizing group purchase of basic living materials, providing door-to-door services for special groups, adjusting the negative emotions of residents due to long-term prevention and control, and stabilizing the normal order of the community. When the number of community workers is stretched thin, their work pressure is extraordinary. Just like the full-time staff of the QXG community committee replied:

*We have been almost automatically canceled weekends and holidays since the outbreak. Because we have to provide nucleic acid testing services for residents every day, we can rest for up to one day on weekends (but we are also arranged to deal with all kinds of emergency affairs). Each of us is physically and mentally exhausted, but there is no personal rest time, nor time to accompany our families, and we have to work continuously day and day. If the pandemic is serious, we usually form a team of three to work overtime for free, and even continue to work in the committee office or the community all night*.

The officer from the JX community police office replied:

*In fact, the police offices in most communities cannot even allocate one police officer and one auxiliary police on average, so some community police officers need to manage two or three communities alone. We are now severely understaffed*.

Similarly, the community hospital with only more than 30 full-time doctors faces a serious shortage of professionals when it encounters an outbreak. The director of the community hospital and the doctor in charge said:

*Basically, during the period when the pandemic first occurred, we may not have time to sleep for several days in a row. At that time, we even had to work until four or five in the morning to complete the relevant emergency response tasks in Z hospital*.

Not only are the community committee, the police office, and the community hospital that directly connects with residents facing enormous pressure, its superior guidance department, the subdistrict headquarters also have a heavy workload. The headquarters and governance principals (municipal and district level) belong to government departments, many of their staff are recruited after the establishment of government institutions, and quota of personnel number or job distribution. Therefore, the government has the power of personnel assessment, promotion, accountability and dismissal of them. In addition, in the process of responding to the pandemic, government departments have also set up a corresponding assessment mechanism, which makes the main leaders at subdistrict level maintain vigilance and pressure on the work for a long time. Deputy Director of pandemic prevention and control headquarters in C subdistrict replied:

*We have a job today that ranks last among 18 subdistricts in H district, so I have been criticized by my superiors for a long time, and I am now frantically looking for a way to remedy it. In addition, I will redesign the prevention and control plan tomorrow and the day after tomorrow (Saturday and Sunday), organize colleagues and subordinates to discuss these on Monday. I have to go back to work overtime tomorrow (Saturday), and at this moment (23:30 pm) I am still consulting other subdistrict leaders who have experienced in emergency response*.

#### 3.2.2. Unclear emergency responsibilities and rights

The community follows an emergency responsibilities and rights mechanism that is linked up and down, and its fundamental purpose is to effectively block the spread of the pandemic. The specific action principals are the local government and community organizations (community committees, owners committees, property management companies, community hospitals, and community police offices, etc.) under the collaborative governance framework. In addition, the main content is to build a multi-stage and whole process community system from “emergency prevention and preparation” to “emergency recovery and reconstruction”. The community committee, as a mass autonomous organization, is not the main actor of administrative law enforcement and generally does not have the capacity to prevent and reduce emergencies. However, facing the dilemma of multiple leaders and overwhelmed, the community committee has always struggled and insisted on the front line in the process of pandemic prevention and control. The full-time staff of the QXG community committee said:

*After the outbreak of pandemic, it can be seen from the work we have done that no matter what tasks the superiors have, they are finally handed over to the community committees. If our committee has a lot of staff, it doesn't matter whether we take on more work or not, but there are only 5 of us, and our colleagues are all sick or fall ill due to overwork without rest for a long time. Sometimes, the police station and community hospital will also assign tasks to us, so that we still don't know how many leaders we have at present*.

Although heavy tasks need to be completed every day, the wages and benefits of staff involved in community emergency governance are relatively low. Just like the feedback from the staff of QXG community committee:

*Since the outbreak of pandemic, our daily workload has greatly increased, but there have been no new benefits and wage increases. Even vacations are automatically canceled due to the demands of the work. Not long ago, a colleague was just transferred from here, and everyone wanted to set off firecrackers to celebrate his escape from suffering*.

Due to the unclear responsibility and rights relationship, the fuzzy responsibility positioning, the insufficient incentive for grassroots workers and the overload operation of the grassroots governance principals, it will inevitably lead to one kind or another of governance problems, and cause the staff participating in community governance to reduce recognition of their own work and show job burnout behavior.

#### 3.2.3. Inadequate emergency warning

Effective community emergency prevention and early warning can reduce the large-scale spread of infectious diseases. In the process of responding to COVID-19, communities have gradually established emergency plans for answering public health events, but the plans usually lack emergency drills or practical tests. In addition, the emergency plan of pandemic prevention and control headquarters in C subdistrict still lacks detailed division of labor of specific departments and personnel. In the process of learning practical experience from surrounding subdistricts, we also found that these grassroots units often form temporary groups according to the actual situation of emergencies, and their relevant emergency preparedness experience has not been sublimated to the experience that can be used for reference and promotion. What's more, the experience of emergency preparedness among different subdistricts is lack of being communicated and shared, so the initiative of the governance principals is insufficient. The Deputy Director of pandemic prevention and control headquarters of C subdistrict said:

*Only the subdistricts that have actually fought against the pandemic will know more about how to design emergency warning plan better. Because we haven't actually experienced a large-scale outbreak, we really want to make a plan now, but we don't know how to design it or design it scientifically*.

#### 3.2.4. Limited emergency participation

Because the virus is infectious, community committees give priority to the participation of professionally trained party members in the voluntary service activities, while ordinary residents are difficult to engaged in the emergency governance of public health events due to the lack of professional training in advance, so can only provide completing some simple voluntary services. The full-time cadre of XT owners' committee said:

*The community committee first organizes volunteers from party member organization to get involved in voluntary service activities when it carries out nucleic acid testing. Only when its volunteer reserves are insufficient, will it consult our owners committee for volunteer backup resources*.

In addition, the community still needs to stimulate the residents' initiative to attend emergency governance. Deputy Director of CT community committee said:

*In a community (especially an old community with many single buildings), each building has a building leader who is responsible for the emergency management. These people are mainly CPC party members. Sometimes there is a situation where no one in the whole building is willing to be the building leader, primarily because the work is public welfare (no salary). It has been almost three years for pandemic prevention and control, so many residents will be no original willingness or enthusiasm to enter this matter as they have their own work*.

Going back to the source, because the education and long-term incentive mechanism for community emergency participation is not yet perfect, it has not been able to fully mobilize the institutions, markets, dwellers and other forces to automatically enroll in the emergency services. Especially in the aspect of professional supports such as medical care, psychology, software, etc., social participation is still very weak, which may easily lead to insufficient hematopoietic function and weak resilience of the community itself.

## 4. Discussion

**Figure 2:**
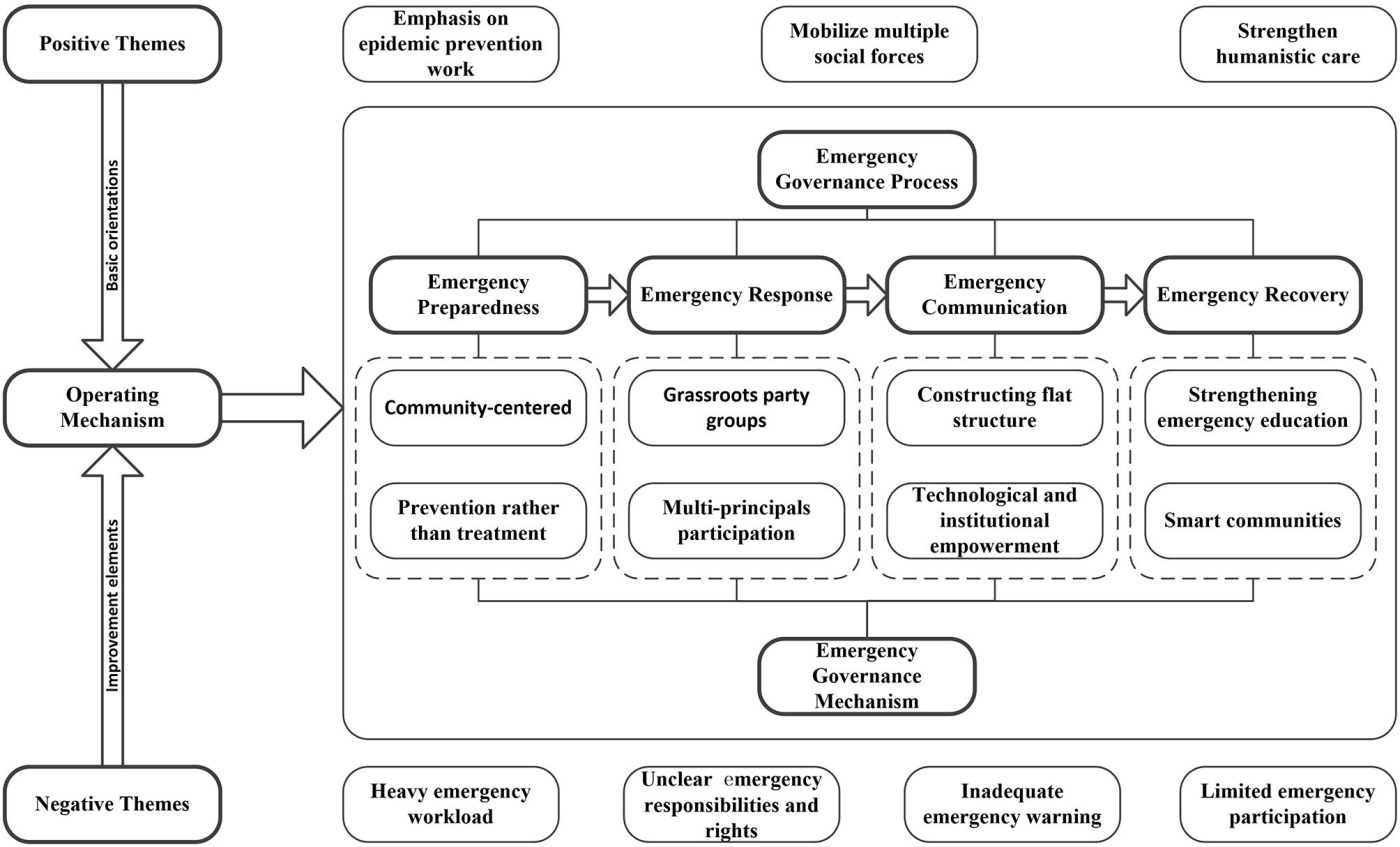
Mechanism innovation of community emergency governance.

The community, as the basic unit of social governance, is the front line of emergency prevention, mitigation and response in Chinese cities. Therefore, the key to unremitting pandemic prevention and control depends on communities. Since the “SARS event” in 2003, China has gradually established emergency management plans, systems and mechanisms, and the governance system has also made great progress (35). Since then, China has begun to implement a comprehensive emergency governance system with traits of “all-risk management,” “multi-principals participation,” and “whole life cycle management” (36). Among them, the 4R Crisis Management Model which includes four stages of reduction, readiness, response and recovery, is usually applied in the whole life cycle management of crisis events (37). As the object of emergency governance has great similarities with the characteristics of public crisis, Robert Health's 4R Crisis Management Model provides a theoretical basis for this study. Therefore, in light of the advantages and dilemmas on community emergency governance in Guangzhou during COVID-19 stage, this study attempts to explore the innovative mechanism of emergency governance in urban communities based on the aspects of preparedness, response, communication and recovery. The mechanism innovation of it is shown in Figure 2.

### 4.1. Innovate preparedness mechanism: Prevention rather than treatment

From 2008 to 2020, China's system and mechanism for managing public health events tended to “emphasize treatment and neglect prevention” (38). However, the COVID-19, a major public health event, is highly infectious, especially the mutant strains such as Omicron, which has strong transmission capacity and concealment. If we continue to adhere to the passive response of “treatment priority”, it is bound to be unable to block the spread of the pandemic from the source. Therefore, the multiple principals of emergency governance in urban communities needs change the concept as soon as possible, and insist on giving priority to prevention in the context of normalization control, so as to avoid large-scale outbreaks as much as possible. (1) Community committees and connected competent agencies can formulate emergency plans based on their actual needs and on the condition that they listen to the opinions of public health experts, superior departments and residents in various aspects. Its contents include: assisting in infectious disease investigation, collecting, processing and reporting information, emergency full-time positions and the responsibilities of their staff, daily information release and feedback methods, communication procedures with health institutions, isolation safeguards measures, and assistance to special groups, etc. (2) Community committees can test the operability of emergency plans by conducting prevention and control drills together with other principals, and finally build an early warning system for public health events which composed of government departments, community governance organizations, residents' families and volunteers, it can continuously improving residents' awareness of health and safety prevention. (3) At present, the COVID-19 is showing a trend of multiple local outbreaks. Therefore, it is necessary to focus on improving the prevention and control capabilities of grassroots principals, such as community committees, police stations, and community hospitals, and then stimulate the vitality of local party members and the masses to energetically participate in community emergency governance. For example, mobilizing residents to positively cooperate with property management companies to complete temperature measurement and disinfection, register personal information by scanning code, check itinerary cards, carry out nucleic acid testing, etc. So in this way, it can speed up the vaccination proceeding of community residents, and comprehensively build a strict line of guard from community joint prevention and control.

### 4.2. Innovate response mechanism: Multi-principals collaborative participation

An important feature that distinguishes “governance” from “management” is that governance principals are more diversified (39, 40). In light of the limitations of government-led direct-control management model, some studies have put forward the construction ideas of community emergency governance from vertical to horizontal, fragmented to collaborative, segmentation to integrity, and formed a diversified collaborative practice mode of “party organization leadership, public participation, and social support” ultimately (41). As members of the community, such as community committees, owners committees, property companies, resident organs, social groups and volunteers etc., are usually in a discrete and weak connection state, it is difficult to spontaneously form coordinated collective actions among them, so they lack the capacity to match these when facing risks (42). In the practice of grassroots governance, the subdistrict headquarters mainly supervises the “three-person team” to complete community emergency tasks, while multiple different principals are important collaborative forces, so they need to give full play to their positive role on community emergency governance and service provision.

First of all, it is essential to refine the rights and responsibilities of governance in the joint prevention and control mechanism of urban communities, clarify the respective institutional settings, rights and responsibilities allocation, and personnel composition of “three-person team”. Ensuring that different organizations perform their own duties, so as to avoid the problem that various organizations at the same level or higher-level departments exert excessive pressure on community committees, which belongs to the mass self-governing organization. Secondly, the government should properly authorize and entrust the management of affairs in specific fields to communities or social agencies, and improve the grassroots emergency capacity through continuous innovation of institutional mechanisms. Thirdly, the government should pay much attention to improve the welfare of principals when participating in governance for better stimulate their enthusiasm on collaborative work. In terms of “three-person team”, it is necessary to appropriately increase the number of staff to alleviate the serious understaffed, and the welfare subsidies or wages to increase members' recognition and sense of belonging to their work. In terms of the enterprises in subdistrict, the right to priority development can be given to community service and poverty alleviation businesses. By formulating enterprises' cultivation and support plans, and building a network platform connecting enterprise supply or community needs, communities can not only get material aid, but also obtain information, capital, technology and other help when dealing with major public health events. As for community inhabitants, the methods include publicity and training can be used to promote their enthusiasm for cooperating with the work of community prevention and control, and arouse mass volunteers to effectively alleviate the trouble of insufficient human resources through establishing professional training and long-term incentive mechanisms. Finally, consolidate the achievements of pandemic prevention and control with humanistic care, especially focus on the elderly and special groups (disabled persons, pregnant women and children, etc.), zealously respond to residents' demands and provide emergency services that are convenient for the masses.

### 4.3. Innovate communication mechanism: Constructing flat organizational structure

In order to enhance the exchange of alerts and information, ensure grassroots organizations and each resident can take corresponding actions in emergencies, the establishment of a communication mechanism for public health events should be set as an important issue. On the one hand, construction and operation of emergency communication mechanism for grassroots governance: we can't just rely on the independent role of a community or organization, but we can consider stimulating different social principals to participate in the governance of public health events at the same time. By sharing pandemic prevention and control information and human, financial or material resources, it is possible to facilitate the whole process governance as soon as possible. Firstly, sharing the public basic database and the emergency information platform of government departments in real time as required, thereby improving the community's overall governance capacity for emergencies. Secondly, promoting the seamless connection between community emergency platform and daily service work, and promote the informatization and grid construction for community emergencies. Thirdly, in accordance with substantive requirements and specific regulations of community prevention and control, actively increase the communication between the community and social organizations, various enterprises and institutions, and strengthen the information technology forces or precise governance. Fourth, for the design of emergency plans, different subdistricts should pay attention to the sublimate experience on pandemic prevention and control in the period of normalization and abnormal state, and the superior departments should organize all subdistricts and communities to learn and exchange emergency preparedness experience, so as to heighten the reference and promotion of advanced emergency plans or experience, and finally upgrade the governance standard of all subdistricts and communities as soon as possible.

On the other hand, construction and operation of emergency communication mechanism for community residents: The emergency communication channels can be unblocked through multi-dimensional methods such as technical and institutional empowerment, in order to boost the initiative of community residents in coping with the pandemic, and realize self-rescue or mutual assistance as well as community micro-governance. Firstly, make full use of internet technology, adopt a combination of online listening and offline mobilization to establish a smooth channel for residents' interest appeal, and strengthen communication with community residents. Secondly, fortify the performance of social autonomy and cultivate highly professional and technical voluntary groups from residents, improve the popular science and non-governmental functions in pandemic prevention and control, so that achieve the health and safety of residents and the stability of economy or society in the post-pandemic era. For example, the community elevates the integral system of settlement, improve the incentive mechanism of the integration on residents' participation channels, autonomy, regulation of law, rule of virtue, and explore the time for inhabitants to take part in community emergency governance as scores and deposit them in the “Public Welfare Time Bank”. Finally, the emergencies prevention awareness and necessary skills of inhabitants should be enhanced to realize the 360-degree governance for community prevention and control.

### 4.4. Innovate recovery mechanism: Building smart communities

The establishment of smart communities is the internal element and external force to ensure community emergency recovery in megacities, which can be carried out mainly by improving the consciousness of community residents to deal with major public health events (internal factors) and the modern information technology (external force) for pandemic prevention and control. In order to broaden residents' emergency knowledge, improve their consciousness, and strength the ability of grassroots staff, emergency education and training is particularly important. First of all, public health experts can carry out community activities focusing on major events related to people's life, health and safety. Such as infectious disease prevention and control knowledge, community emergency management, and the use of medical protective equipment, so that the community can better play the role of grassroots sentinel, earnestly pay close attention to a series of measures on “external anti-input, internal anti-rebound”, then effectively improve the level of grassroots governance. Secondly, continue to carry out professional training for community cadres, grid leaders, members of the “two committees”, property management personnel, and volunteers, so that they can fully realize the importance and sense of mission on emergency work, deepen their understanding of policies and laws, and accurately implement the prevention and control requirements of superior. Finally, take the family as a unit to execute emergency education and publicity for residents. Especially the channel includes community official accounts, WeChat owners groups, electronic bulletin boards, Douyin videos, propaganda banners, paper leaflets or handbooks, etc. These can be used to instruct residents to hold ventilate, wash hands frequently, wear masks, inoculate vaccinate, maintain social distance, and persist in physical exercise to enhance disease resistance.

The emergency recovery work is also carried out closely around modern information technology. The community can make full use of emerging tools such as Big Data and artificial intelligence to strengthen the dynamic tracking management of emergency information, so as to achieve the triple effect of civil air defense, technical and physical defense. In the process of COVID-19 prevention and control, many internet companies have demonstrated superb scientific and technological achievements, such as the “Pandemic Information Collection and Management System” built by Alibaba Cloud, the “Migration Big Data Map” developed by Baidu Company, and the “Travel Path Query Service” launched by three major operators (China Unicom, China Mobile, and China Telecom), which have made major contributions to efficient implementation of pandemic prevention and control. Therefore, on the basis of comprehensive service platform, the integration of Big Data, cloud computing, and artificial intelligence means it can complete the rapid connection and conversion of normalized state for prevention and control. In addition, smart devices such as AI face capture and recognition cameras, non-contact infrared thermometers, and voiceprint data collection terminals are used to improve the non-contact level of community governance and control services, thereby improving the efficiency of community governance. What's more, break down departmental and regional barriers and built a data resource sharing system with unified command, interconnection, security and reliability (22, 43, 44). So we can improve community smart service systems, medical care, and elderly applications to create a “convenient service circle with shortest distance” ultimately.

## 5. Conclusion

The current research on community emergency governance focuses on the response and management of natural disasters, but the governance support for public health events is relatively rare (16, 18, 27). Therefore, based on the real status of pandemic prevention and control in China, this study further broadens the research scope of grassroots emergency governance. In light of the current important position and influence of urban community organizations in the process of normalized control, this study emphasizes leading collaborative governance development of multiple principals by grassroots party groups, building smart community, strengthening humanistic care, and demonstrating human warmth of community, etc., which gives different application values to ensure the bottom line of persons' livelihood on public health events. How to scientifically respond to public health events is not only closely related to individual citizens, but also to social stability and sustainable economic development. In the post-pandemic era, this study proposes to innovate the governance mechanism from the basic orientations of positive practice and the improvement elements from negative practice faced by Guangzhou community in response to pandemic.

It is worth mentioning that the top-down response to the outbreak in Guangzhou and other cities in China has turned out to be effective under China's current administrative system. Therefore, the paper conducts an in-depth discussion on how to innovate public health emergency mechanism in urban communities. Unfortunately, due to the remarkable top-down characteristics of China's prevention and control operations, community residents rarely participate in community governance as individuals. Therefore, this research focuses on qualitative study from the perspective of grassroots organizations rather than individuals, and does not pay enough attention to community inhabitants. So, it is hoped that subsequent research can make up for the above shortcomings through quantitative or comparative study.

## Data availability statement

The datasets presented in this study can be found in online repositories. The names of the repository/repositories and accession number(s) can be found in the article/supplementary material.

## Ethics statement

Ethical review and approval was not required for the study on human participants in accordance with the local legislation and institutional requirements. Written informed consent from the participants was not required to participate in this study in accordance with the national legislation and the institutional requirements.

## Author contributions

LZ designed the study, conducted field research, and drafted manuscript. FO participated in the process of field research and manuscript drafting and editing. All authors contributed to the article and approved the submitted version.
